# CONCERNED AND LONELY AMIDST COVID-19: FINANCIAL STRAIN AND LATER-LIFE PSYCHOLOGICAL WELL-BEING IN NEVADA

**DOI:** 10.1093/geroni/igad104.1890

**Published:** 2023-12-21

**Authors:** Tirth Bhatta, Dante Miller, Nirmala Lekhak

**Affiliations:** University of Nevada, Las Vegas, Las Vegas, Nevada, United States; University of Nevada Las Vegas, Las Vegas, Nevada, United States; University of Nevada, Las Vegas, Las Vegas, Nevada, United States

## Abstract

**Objectives:**

Nevada has some of the nation’s highest suicide rates among older adults, which are linked to social isolation, loneliness, and mental health. The COVID-19 pandemic is likely to have exacerbated the loneliness and financial insecurity that older Nevadans have already been experiencing to a greater extent since the 2008 financial crisis. Prior research based on national data has documented the relationship between financial strain and mental health in later life. However, the social and psychological mechanisms that underlie such relationships have not been investigated, particularly in light of the devastating economic and health effects of the pandemic on older adults in Nevada.

**Methods:**

Based on our web-based survey of adults aged 50 years and older (n=313), we examined the roles of loneliness and concerns about the pandemic in explaining the impact of financial strain on depressive symptoms and anxiety.

**Results:**

We documented a statistically significant effect of financial strain on depressive symptoms (b=2.41, p-value< 0.001) and anxiety (b=1.74, p-value< 0.001). The inclusion of loneliness and concerns about the pandemic in our regression models led to substantial reduction in the impact of financial strain on depressive symptoms, whereas such impact on anxiety became statistically not significant. We also found significantly higher levels of depressive symptoms and anxiety among our respondents who reported greater feeling loneliness and concerns about the pandemic.

**Discussion:**

Our findings underscore the need for mental health resources and financial support programs for economically disadvantaged older adults to alleviate their feelings of loneliness and improve mental health in Nevada.

